# Potential pathogenic mechanism of type 1 X-linked lymphoproliferative syndrome caused by a mutation of *SH2D1A* gene in an infant: A case report

**DOI:** 10.1097/MD.0000000000030951

**Published:** 2022-10-14

**Authors:** Yanchun Wang, Yan Wang, Weimin Lu, Lvyan Tao, Yang Xiao, Yuantao Zhou, Xiaoli He, Yu Zhang, Li Li

**Affiliations:** a Second Department of Infectious Disease, Kunming Children’s Hospital, Kunming, Yunnan, China; b Kunming Key Laboratory of Children Infection and Immunity, Yunnan Key Laboratory of Children’s Major Disease Research, Yunnan Institute of Pediatrics, Yunnan Province Clinical Research Center for Children’s Health and Disease. Kunming Children’s Hospital, Kunming, Yunnan, China; c Department of Otorhinolaryngology Head and Neck surgery, Kunming Children’s Hospital, Kunming, Yunnan, China.

**Keywords:** AKT, mTOR, PI3K, SAP, *SH2D1A*, X-linked lymphoproliferative syndrome

## Abstract

X-linked lymphoproliferative syndrome (XLP) is a rare X-linked recessive inborn errors of immunity. The pathogenesis of XLP might be related to phophatidylinositol-3-kinase (PI3K)-associated pathways but insight details remain unclear. This study was to study an infant XLP-1 case caused by a mutation in *SH2D1A* gene, investigate the structural and functional alteration of mutant SAP protein, and explore the potential role of PI3K-associated pathways in the progression of XLP-1.

**Methods:**

The proband’s condition was monitored by laboratory and imagological examinations. Whole exome sequencing and Sanger sequencing were performed to detect the genetic disorder. Bioinformatics tools including PolyPhen-2, SWISS-MODEL and SWISS-PDB Viewer were used to predict the pathogenicity and estimate structural change of mutant protein. Flow cytometry was used to investigate expression of SAP and PI3K-associated proteins.

**Results:**

The proband was diagnosed with XLP-1 caused by a hemizygous mutation c.96G > T in *SH2D1A* gene resulting in a missense substitution of Arginine to Serine at the site of amino acid 32 (p.R32S). The mutant protein contained a hydrogen bond turnover at the site of mutation and was predicted to be highly pathogenic. Expression of *SH2D1A* encoded protein SAP was downregulated in proband. The PI3K-AKT-mTOR signaling pathway was fully activated in XLP-1 patients, but it was inactive or only partially activated in healthy people or HLH patients.

**Conclusions:**

The mutation c.96G > T in *SH2D1A* gene caused structural and functional changes in the SAP protein, resulting in XLP-1. The PI3K-AKT-mTOR signaling pathway may play a role in XLP-1 pathogenesis.

## 1. Introduction

X-linked lymphoproliferative syndrome (XLP) is an extremely rare X-linked recessive inborn errors of immunity caused by genetic variations, with a 1/1000000 incidence rate,^[12]^. XLP is currently classified into 2 forms based on mutant genes: Type 1 XLP (XLP-1) and Type 2 XLP (XLP-2) caused by mutations in *SH2D1A* gene and *XIAP* gene, respectively^[3,4]^. Common clinical characteristics of XLP-1 and XLP-2 including Epstein-Barr virus (EBV) infection, hemophagocytic lymphohistiocytosis (HLH), lymphoproliferative disorder, and dysgammaglobulinemia, but sometimes they also manifested differently. According to published studies, EBV infection and associated HLH were more frequently observed in patients with XLP-1, and sometimes even accompanied by catastrophic neurologic disorders. Development of lymphoma is only reported in XLP-1 patients^[5]^. Moreover, Natural killer cells (NK) and T lymphocytes are more likely to proliferate and infiltrate organs, including liver, spleen, and lymph nodes, in EBV-infected XLP-1 patients^[6]^. Persistent hypogammaglobulinemia appeared more often in XLP-1 patients while transient hypogammaglobulinemia was more frequently observed in XLP-2^[7]^. Inflammatory bowel disease, such as Crohn’s disease, was exclusively discovered in XLP-2 patients, and roughly 25% to 30% of XLP-2 patients were impacted^[8]^. Additionally, fever, subcutaneous petechia, hematemesis, hematochezia, pistaxis, jaundice, hepatosplenomegaly, lymphadenopathy, lymphocytic vasculitis, aplastic anemia, and lymphomatoid granulomatosis may also be signs and symptoms of XLP^[9–11]^. Due to the rapid progression and complicated symptoms of XLP, it is commonly misdiagnosed clinically. XLP has a poor prognosis and high mortality, which have been reported to be 75%, of which 70% died before the age of 10^[12]^. At present, the only promising treatment is allogeneic hematopoietic stem cell transplantation (HSCT) before the onset of typical symptoms or EBV infection^[13]^.

The *SH2D1A* gene is found on chromosome Xq25 and has 4 exons, and mutations in this gene can result in SAP protein deficiency. SAP is a 128-amino acids signaling lymphocyte activating molecule (SLAM)-associated protein that contains 1 Src homology 2 (SH2) domain and is primarily expressed in T cells, NK cells, and some EBV-positive Burkitt lymphoma-derived B cells.^[14]^ SAP can competitively bind to SLAMs via the SH2 domain and regulate a variety of processes, including the development and function of invariant natural killer T cells (iNKT), the clearance of EBV-infected B cells with cytotoxic T lymphocytes (CTLs) and NK cells, the development of germinal centers, the production of immunoglobulin, T cell restimulation-induced cell death, and the maintenance of T cell homeostasis.^[15]^ In XLP-1 patients, SAP deficiency can prevent CTLs and NK cells from killing EBV-infected B cells.^[16]^ The *XIAP* gene is also located on chromosome Xq25 and includes 7 exons, its encoding protein XIAP is a novel member of the inhibitor of apoptosis proteins family, which inhibits caspases directly and regulates apoptosis through several routes. *XIAP* gene is overexpressed in many tumor cell lines, and its expression is closely related to tumor progression, recurrence, prognosis, and treatment resistance.^[17]^ Phophatidylinositol-3-kinase (PI3K) is a crucial signaling center in immune cells that catalyzes the conversion of PIP2 to PIP3 and thus facilitates membrane recruitment of molecules containing PIP3-binding Pleckstrin homology domains such as the AKT, PDK1, Tec family kinases, adapter molecules, guanine nucleotide exchange factors, and GTPase-activating proteins. These subsequently activate a number of important downstream pathways including mTOR, FOXO1 and BACH2-related pathways.^[18]^ Too little or too much activity in the PI3K downstream pathway is harmful even pathogenic. Previous research discovered that SAP protein was downregulated in CTLs while the SLAM protein 2B4 was upregulated in patients with congenital PI3K deficiency disorder such as activated PI3Kδ syndrome, implying that SAP may interact with PI3K-associated pathways and XLP-1 may be related to PI3K signaling failure.^[19]^ Insight details of the interaction patterns between SAP and PI3K, as well as the role of PI3K-associated pathways in pathogenesis of XLP-1 still need to be investigated further.

In this study, we will describe an infant with XLP-1, assess the proband’s clinical features and genetic variants, analyze the structural and functional changes in SAP protein caused by gene mutation, and investigate the probable role of PI3K-associated pathways in XLP-1 pathogenesis.

## 2. Methods

### 2.1. Editorial policies and ethical considerations

The study was approved by the Ethics Committee of Kunming Children’s Hospital. All experiments were performed in compliance with the Helsinki Declaration. Informed written consent was obtained from the parents of the proband for the collection of clinical information, blood samples, DNA and presentation of patient’s materials.

### 2.2. Proband

The proband, a 7-month-old male, was admitted to the hospital due to “fever with rash.” Fever was up to 40°C and not relieved after treatment. Physical examination of the proband on admission observed listless and poor mental response, scattered rashes on the trunk and limbs particularly on both forearms and lower legs, eyelid edema, pharyngeal congestion, white pustules attached to the pharyngeal tonsils, and several palpable enlarged lymph nodes in the neck up to about 1 × 1 cm in size with medium hardness and poor range of motion. The proband was initially treated with ganciclovir, sodium succinate and intravenous IgG to against viral infection, reduce inflammation and adjust immunity, respectively. With the progression of the disease, the proband had recurring fevers and icteric sclera indicating aggravated liver damage. The proband’s liver and spleen were progressively enlarged, and the rash became more widespread and fusing into patches in some area. *Staphylococcus aureus* were tested positive in sputum. Cefperazone-Sulbactam, meropenem and vancomycin were successively given against infection. Bone marrow examination showed hemophagocytosis. Etoposide was then given, during the treatment, the proband experienced progressive pancytopenia and was interfered with the infusion of granulocyte colony-stimulating factor, interleukin-11, and apheresis platelets. Unfortunately, the proband’s condition kept aggravating despite accepting treatment and finally died due to multiple organ dysfunctions. Parents of the proband were healthy, and no other remarkable diseases history was identified in the family.

### 2.3. Whole exome sequencing

Peripheral blood samples were collected from the proband and parents. Genomic DNA was extracted from peripheral blood using the DNA Extraction kit (CWBIO, China). The isolated DNA was quantified by agarose gel electrophoresis and Nanodrop 2000 (Thermal Fisher Scientific). Libraries were prepared using Illumina standard protocol and a minimum of 3 μg DNA was employed for the indexed Illumina libraries. To capture targeted genes, the biotinylated capture probes were designed to tile all of the exons with non-repeated regions. The resulting captured DNA was amplified by PCR and the PCR product was purified with SPRI beads (Beckman Coulter). The enriched libraries were sequenced on an Illumina NextSeq 500 sequencer for paired-end reads of 150 bp. Quality control criteria were applied to the raw sequencing data before being mapped to the UCSC hg19 human reference genome using BWA (0.7.10). Picard tools (1.119) were used to eliminate duplicated reads. SNP and InDels were detected and filtered by GATK (Genome Analysis TK-3.3.0). Variants were further annotated by ANNOVAR. All mutations identified were confirmed by Sanger sequencing. Sites of variation were identified through a comparison of DNA sequences with the corresponding GenBank (www.ncbi.nlm.nih.gov) reference sequences. Exon 1 of *SH2D1A* (NM_002351) were amplified using 2 pair primers (Forward: 5’-GCTCGATCGAACCAAGCTAC-3’; Reverse: 5’-GGAGCGAAGGTAAACTGTGG-3’). The PCR samples were visualized on agarose gels, purified and sequenced using the terminator cycle sequencing method on an ABI PRISM 3730 genetic analyzer (Thermo Fisher Scientific). The sequencing results were analyzed using the DNASTAR (Madison) software.

### 2.4. Flow cytometry

Peripheral blood samples were collected from the proband and parents, as well as a familial HLH patient and a healthy child as control. Peripheral blood mononuclear cells were isolated using lymphocyte separation solution. Protein expression in peripheral blood mononuclear cells was determined via flow cytometry. Briefly, cells were fixed and permeabilized sequentially with an IntraPrep Permeabilizaton Reagent kit (Beckman Coulter，A07803) following manufacturer’s instruction. Specific antibodies were then added for incubation overnight, followed by 30 mins incubation with a fluorescently labeled secondary antibody. The flow cytometry tests were carried out with a BD FACSCanto II flow cytometer, and the data was processed with CytExpert 2.0 (Beckman Coulter) software. Antibodies used were listed as follows: Anti-SH2D1A/SAP (Abcam, ab109120); Anti-PI3 Kinase p110δ (Abcam, ab109006); Anti-PI3 Kinase p85α (Abcam, ab191606); Anti-PTEN (Abcam, ab32199); Anti-AKT (CST, 9272s); Anti-phospho-AKT (CST, 9271s); Anti-mTOR (Abcam, ab2732).

### 2.5. Prediction of the pathogenicity and structure of mutant protein

Pathogenicity of mutant protein was predicted by PolyPhen-2 online scoring tool (http://genetics.bwh.harvard.edu/pph2/index.shtml). The greater the pathogenicity, the closer the score is to 1.0. The structure of wild-type protein was created by SWISS-MODEL online tool (http://swissmodel.expasy.org/) and the structure of mutant protein was estimated by SWISS-PDB Viewer 4.1.0 software.

## 3. Results

### 3.1 Laboratory and imagological examinations indicated that the proband’s condition continued to deteriorate despite receiving treatment

**Figure 1.**
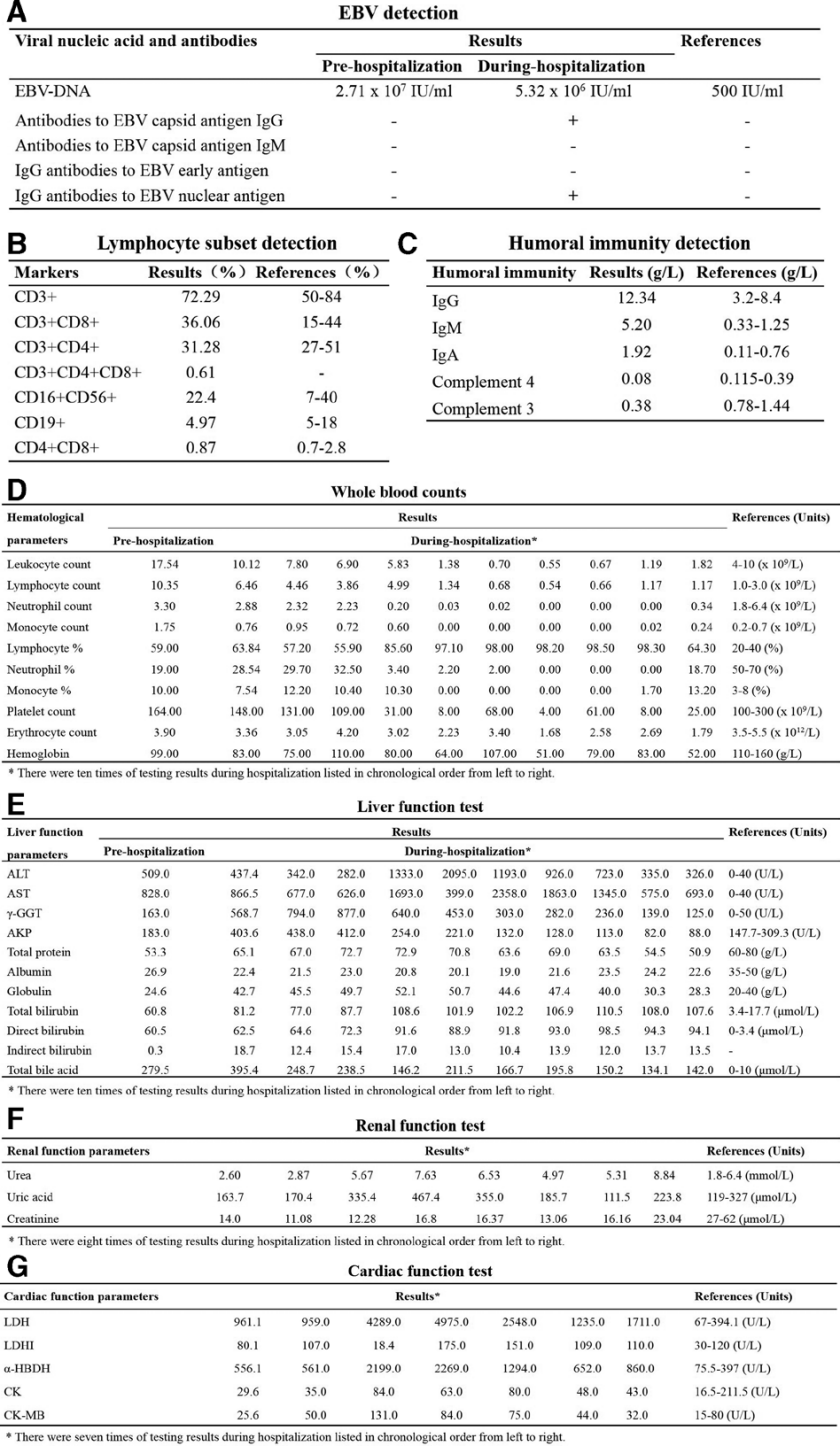
Laboratory examinations of the proband with the progression of disease. (A) Results of peripheral blood EBV nucleic acid and antibodies test. (B) Results of lymphocyte subsets test. (C) Results of humoral immunity test. (D) Results of whole blood counts. There were ten times of testing results during hospitalization listed in chronological order from left to right. (E) Results of liver function test. There were 10 times of testing results during hospitalization listed in chronological order from left to right. (F) Results of renal function test. There were 8 times of testing results during hospitalization listed in chronological order from left to right. (G) Results of cardiac function test. There were 7 times of testing results during hospitalization listed in chronological order from left to right. EBV = Epstein-Barr virus.

**Figure 2.**
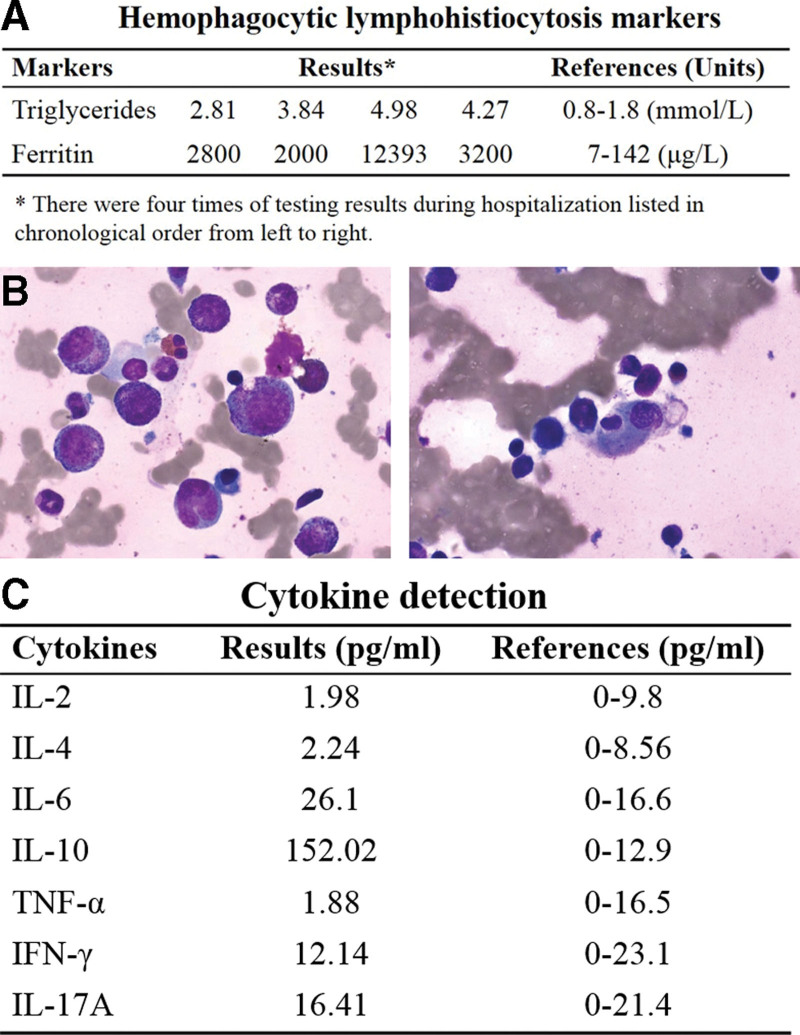
Occurrence of hemophagocytic lymphohistiocytosis was detected in the proband. (A) Results of ferritin and triglycerides tests. There were 4 times of testing results during hospitalization listed in chronological order from left to right. (B) Results of cytopathology detection of bone marrow. (C) Results of serum cytokine test.

**Figure 3.**
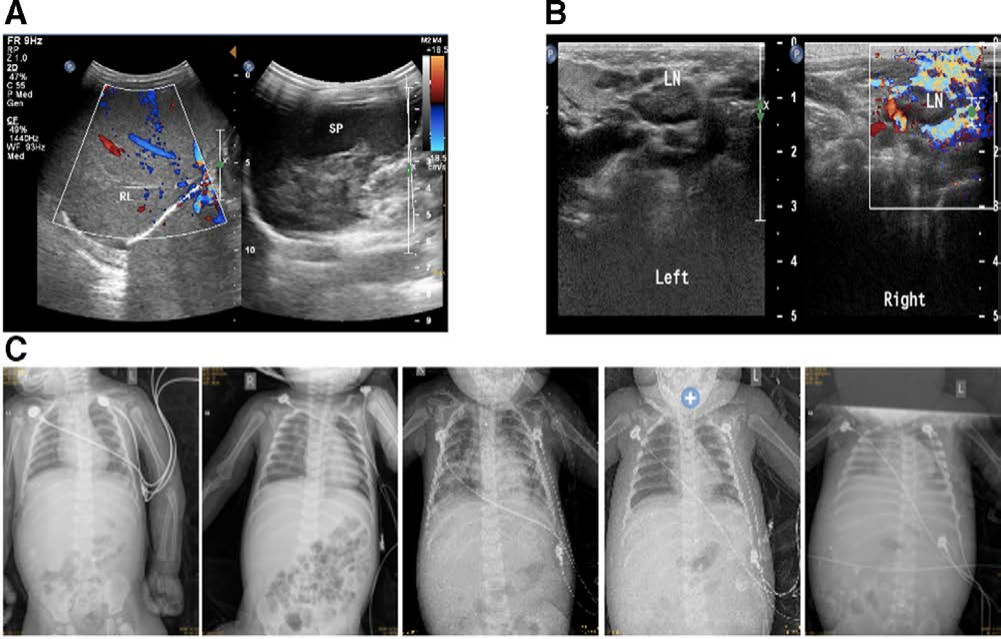
Imageological examinations of the proband with the progression of disease. (A) Results of abdominal color ultrasound. (B) Results of neck color ultrasound. (C) Results of chest X-ray. There were 5 times of examine results during hospitalization listed in chronological order from left to right.

To access detailed conditions of the proband, multiple laboratory and imagological examinations were conducted during hospitalization. Results showed that peripheral blood EBV nucleic acid load continued to rise and the antibodies to EBV capsid antigen (EBVCA) IgG and IgG antibodies to EBV nuclear antigen became positive as the disease progressed (Fig. 1A). The percentage of CD19 + B cells was slightly decreased (Fig. 1B), and humoral immunity test indicated increased levels of serum IgG, IgM, IgA, and decreased C3 (Fig. 1C). Hematological parameters of the proband pre- and during-hospitalization showed that the counts of leukocyte, lymphocyte, neutrophil, monocyte, platelet, erythrocyte and hemoglobin were declined with fluctuation and generally lower than reference values. The percentages of lymphocyte, neutrophil and monocyte were all fluctuated outside the reference value (Fig. 1D). Besides, indicators for liver, renal and cardiac function of proband were abnormal as disease progressed. The levels of alanine aminotransferase, aspartate aminotransferase, γ-glutamyltransferase (γ-GGT), alkaline phosphatase, total bilirubin, direct bilirubin, total bile acid, lactate dehydrogenase and α-hydroxybutyrate dehydrogenase (α-HBDH) were all extremely higher than reference values (Fig. 1E–G). Additionally, the levels of ferritin and triglycerides elevated dramatically indicated the development of HLH (Fig. 2A). Occurrence of HLH was also confirmed by hemophagocytes in bone marrow (Fig. 2B) and increased expression of serum IL-6 and IL-10, with IL-10 being 12 times higher than the reference values (Fig. 2C). Abdominal color ultrasound revealed hepatomegaly, gallbladder wall thickening, mild splenomegaly and abdominal effusion (Fig. 3A). Neck color ultrasound revealed slightly enlarged lymph nodes on both sides of neck and jaw (Fig. 3B). Chest X-ray revealed increased bilateral pleural effusion with deterioration of disease (Fig. 3C). Overall, with the progression of disease, the condition of the proband kept deteriorating, especially the infection was aggravated, immunity responses were deficient, and multiple organs were progressively disabled. The clinical features including EBV-infection, bone marrow hemophagocytosis, dysgammaglobulinemia, hepatosplenomegaly and lymphadenopathy observed suggested that the proband may suffer from XLP.

### 3.2. The proband was diagnosed with XLP-1 caused by a hemizygous mutation c.96G > T in *SH2D1A* gene

**Figure 4.**
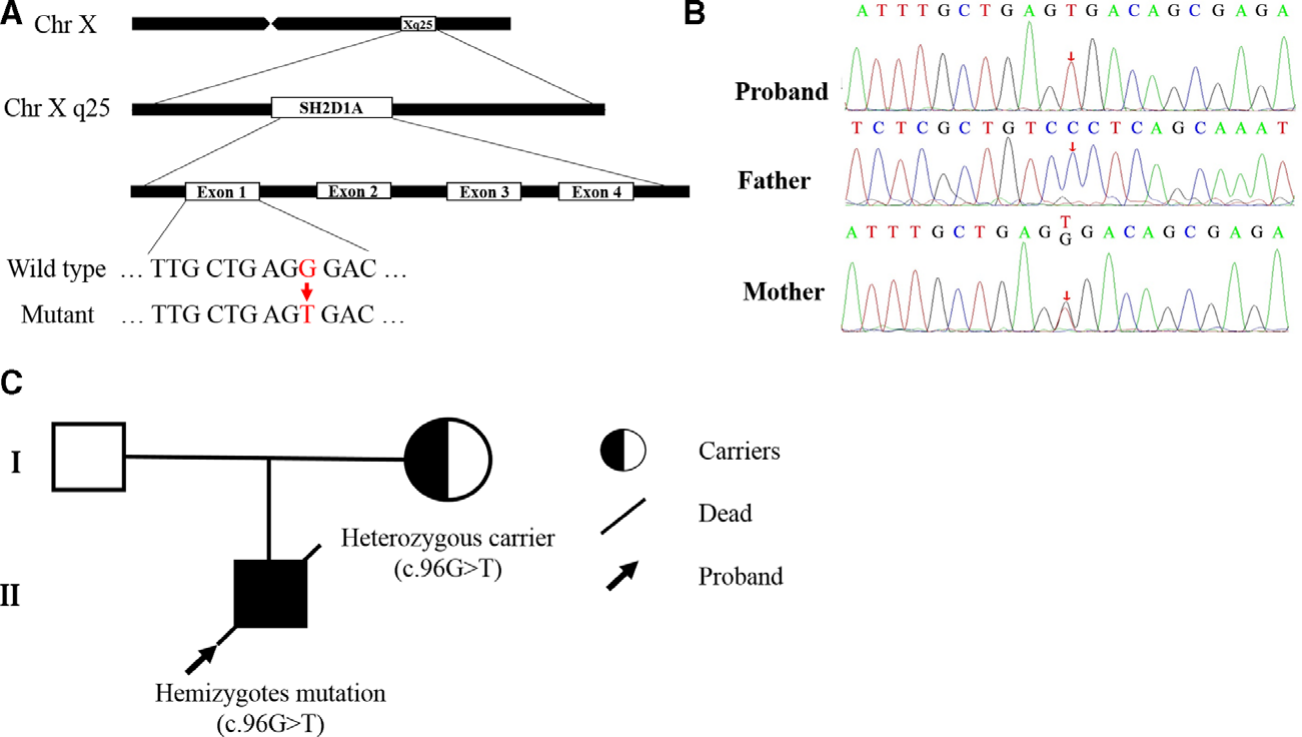
Identification of a hemizygous mutation c.96G > T in SH2D1A gene of the proband. (A) Schematic diagram of SH2D1A gene. The mutation site was highlighted in red. (B) Results of Sanger sequencing. The arrows indicated the sites of mutation. (C) Genetic pedigree of the family.

In order to confirm whether the proband was suffered from genetic disorders, the peripheral blood DNA of the proband and parents were extracted, and whole exome sequencing was performed. The results revealed a hemizygous mutation in exon 1 of *SH2D1A* gene, which substituted Guanine to Thymine at the site of nucleotide 96 (c.96G > T) (Fig. 4A), resulting in a missense substitution of Arginine to Serine at the site of amino acid 32 (p.R32S). According to the ACMG guidelines, this mutation was identified as a pathogenic mutation. It was located in the mutation hotspot region with a low-frequency in the normal population database. The mutation with the same amino acid change as this proband had been reported previously but with different nucleotide change.^[20]^ Sanger sequencing validation of this mutation was performed on the parents, and the results identified no variation at this site in the father and a heterozygous variation at this site in the mother (Fig. 4B and C). As XLP-1 inherited in a recessive pattern, the parents of the proband were all appeared healthy. Therefore, the diagnosis of XLP-1 was confirmed based on the proband’s typical clinical manifestations and genetic analysis results.

### 3.3. The mutant SAP protein was highly pathogenic with structural and functional change

**Figure 5.**
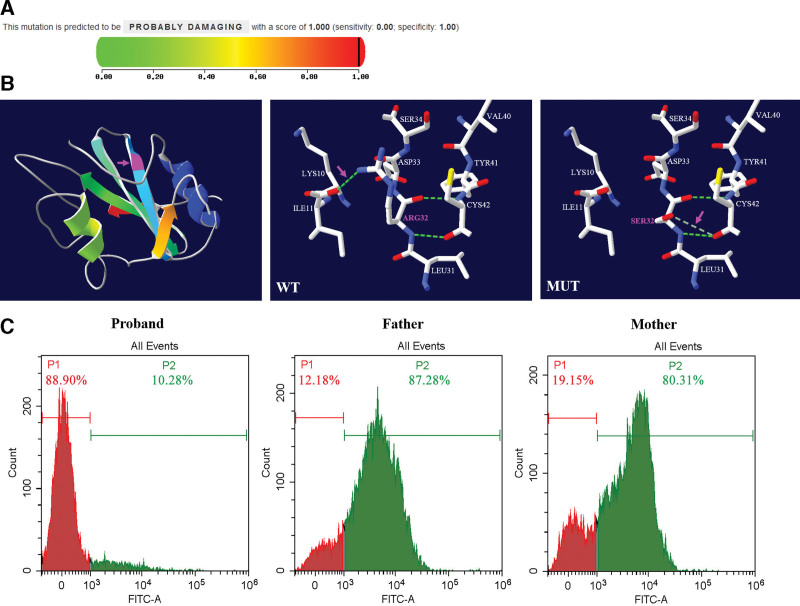
The mutant SAP protein was highly pathogenic with structural and functional. (A) The pathogenicity of mutant SAP protein was predicted by PolyPhen-2. The greater the pathogenicity, the closer the score is to 1.0. (B) The secondary structure of SAP protein and a structural comparison between wild-type and mutant SAP protein. The site and labels of mutation were highlighted in magenta. The green dotted lines illustrated hydrogen bonds. The magenta arrows indicated the turnover of a hydrogen bond. (C) Expression of SAP in the patient and parents detected by flow cytometry. Peaks in red were negative controls. Peaks in green illustrated expression of SAP.

In order to explore whether the c.96G > T mutation in *SH2D1A* gene caused functional or structural deficiency in SAP protein, we first analyzed pathogenicity of mutant protein by PolyPhen-2 online scoring tool. As the greater the pathogenicity, the closer the score is to 1.0, the mutation was predicted to be highly pathogenic with a score of 1.0 (Fig. 5A). In addition, we analyzed the structural change of mutant SAP protein. As shown in Figure 5B, the secondary structure of SAP protein contained 2 α-helices and 8 β-strands and the mutation site was located at the end of the second β-strand. By comparing the structures of wild-type and mutant SAP proteins, we found that the substitution of amino acid Arginine to Serine causing a turnover of a hydrogen bond (Fig. 5B), suggesting the structure of SAP protein was changed. We also detected the expression of SAP in proband and the parents. As expected, flow cytometry detected that SAP protein was significantly downregulated in proband compared to which in parents. As shown in Figure 5C, SAP expression level in the proband, father and mother were 10.28%, 87.28%, and 80.31%, respectively, suggesting the function of SAP in proband was deficient. Overall, c.96G > T mutation of *SH2D1A* gene in the proband led to significant structural and functional deficiency of SAP protein, which was highly pathogenic.

### 3.4. Activation of PI3K-AKT-mTOR signaling pathway in the proband

**Figure 6.**
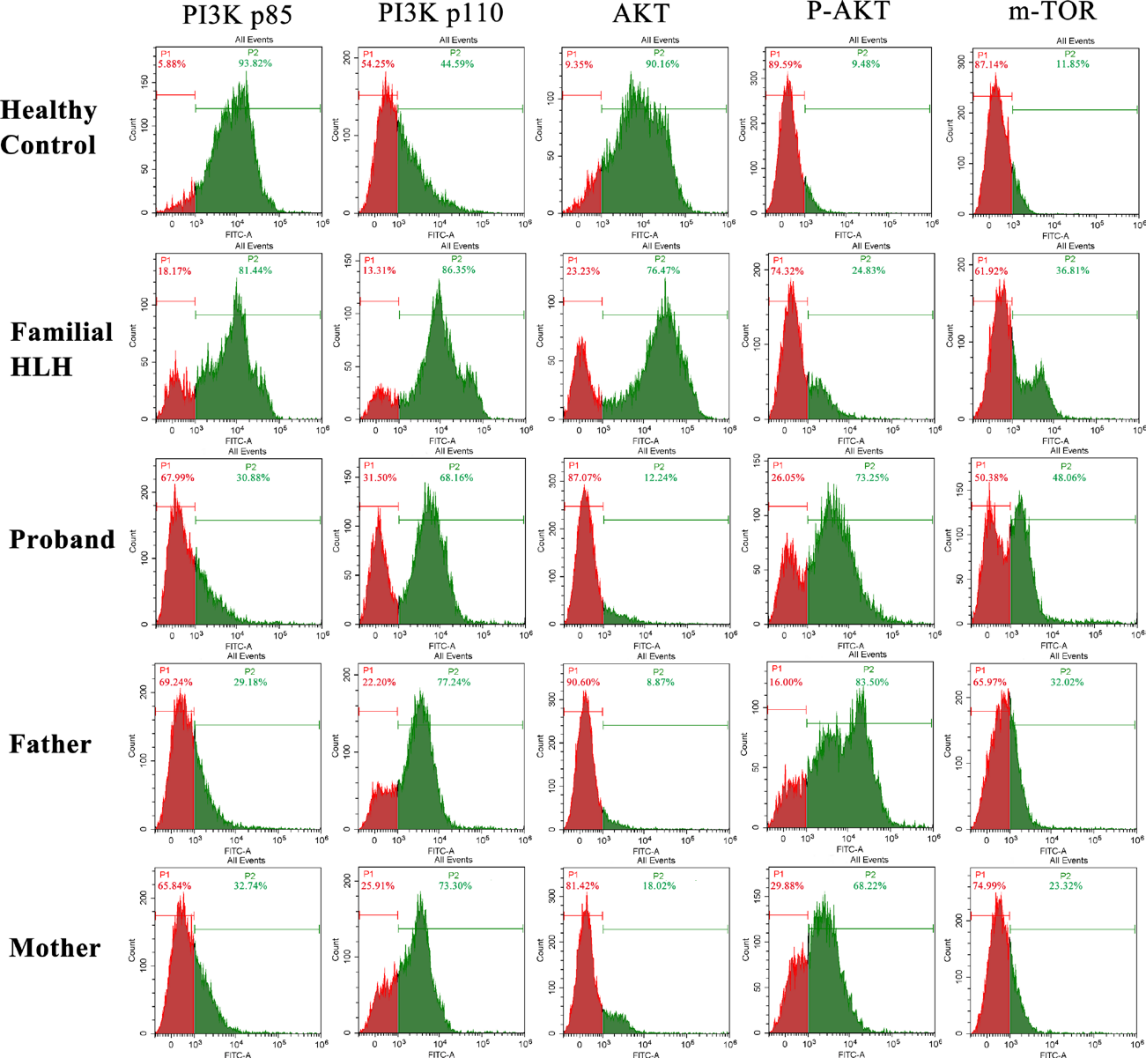
Expression of key proteins in PI3K downstream pathways. Peaks in red were negative controls. Peaks in green illustrated expression of proteins. PI3K = phophatidylinositol-3-kinase.

As previously stated, PI3K downstream signaling pathways may be altered in XLP-1 patients, thus we evaluated the expression of PI3K subunits p110δ and p85α, as well as downstream key proteins including AKT, p-AKT and mTOR in both the proband and the parents. To ensure that observed differences were due to XLP-1 rather than HLH, we also evaluated the expression of proteins in a familial HLH patient and a healthy child. As shown in Figure 6, PI3K-AKT-mTOR pathway was inactivated in the healthy control, as evidenced by high expression of the negative regulatory subunit p85α, which inhibit the activity of the functional subunit p110δ. Moreover, under normal circumstances, the downstream protein AKT was unphosphorylated and mTOR was not expressed. The proband had p85α downregulation and p110δ upregulation, AKT phosphorylation, and mTOR activation, indicating that the PI3K-AKT-mTOR pathway was considerably activated. The expression patterns of p85α, p110δ, AKT, and p-AKT were identical in the father and mother, but mTOR expression was muted. Although p85α and p110δ were excessively high in the familial HLH control, downstream AKT was unphosphorylated and mTOR was only expressed, in contrast to the fully activated PI3K-AKT-mTOR pathway in the XLP-1 proband. Overall, the findings showed that the PI3K-AKT-mTOR signaling pathway was fully activated in XLP-1 patients, but it was inactive or only partially activated in healthy people or HLH patients, implying that the PI3K-AKT-mTOR signaling pathway may play a role in XLP-1 pathogenesis.

## 4. Discussion

Due to complex clinical characteristics, XLP-1 is easily confused with other diseases such as jaundice, hyperbilirubinemia, hypogammaglobulinemia, and lymphoma at the time of diagnosis. Through whole exome sequencing, we identified a hemizygous mutation c.96G > T (p.R32S) in exon 1 of *SH2D1A* gene in the proband of our study, which was inherited from the mother who was a healthy heterozygous carrier of this mutation. Although the sequencing technology greatly assists in the clinical diagnosis and classification of XLP, there is an urgent need for a way to rapidly diagnose XLP due to the acute onset and rapid progression of XLP, especially in cases accompanied by life-threatened HLH. Flow cytometry can be used to quickly detect the expression of SAP or XIAP protein and can also be used to identify the phenotypic and functional deficiency characteristics of lymphocytes in XLP patients, supporting the rapid screening need of XLP. Rapid detection by flow cytometry allows for more treatment time for critical XLP patients and more preparation time for patients suitable for HSCT, which will help improve XLP survival. In this study, we confirmed the SAP protein deficiency in the proband and analyzed the characteristics of peripheral blood lymphocyte subsets via flow cytometry. Based on the clinical manifestations, sequencing and protein expression findings of the proband, we confirmed the diagnosis of XLP-1. Apart from this, flow cytometry can also be used to detect the level of NKT cells which can assist in XLP-1 diagnosis. Previous studies have found that iNKT cells have a constant TCRα receptor and they cannot develop without SAP protein, thus iNKT cells will not be detected in XLP-1 patients when SAP protein is deficient.^[21,22]^ Additionally, iNKT can be labeled with CD3, TCRVα24 and TCRVβ11 antibodies that offer convenience in the detection.^[23]^ Overall, combination of sequencing technology and flow cytometry provides a more efficient and accurate way in the diagnosis and classification of XLP.

SAP protein is an adaptor molecule, which competitively binds to the intracellular region of the SLAM receptor proteins such as 2B4 (SLAMF4) and Ly108 (NTB-A or SLAMF6).^[24]^ The SLAM proteins are particularly important in the generation of cytotoxic effects. In the lack of SAP, the SLAM proteins play inhibitory roles by binding to their ligands and recruiting phosphatases including SHP-1, SHP-2 and SHIP1.^[25]^ Those inhibitory activities are easily triggered in B cells since high levels of SLAM proteins and their ligands are expressed in B cells.^[19]^ Previous research found that the typical cytological characteristics of XLP-1 are functional deficiencies in CTLs and NK cells, which preventing them from killing EBV-infected B cells but retaining the ability to kill many other targets.^[16]^ Moreover, T cell restimulation-induced cell death was disabled possibly due to the inhibitory effect produced by SLAM proteins and their ligands in XLP-1 patients, hence catastrophic lymphoproliferation and HLH were very likely to be induced in the presence of EBV infection.^[26]^ The pathogenic mechanisms of XLP-1 have not been fully understood so far.

Interestingly, we found that the PI3K-AKT-mTOR signaling pathway was fully activated in XLP-1 patients, but it was inactive or only partially activated in healthy people or HLH patients. It is known that PI3K signaling pathways are closely related to lymphocyte development and differentiation.^[27]^ In congenital PI3K deficiency disorders such as activated PI3Kδ syndrome, its over-activation can block B cell development and defect class switching of antibodies, resulting in the production of low affinity antibodies and high expression of IgM.^[28]^ In this study, the proportion of CD19 + B cells of the proband was slightly lower than the reference value, suggesting that the differentiation and amplification of mature B cells may be defective after the activation of PI3K-AKT-mTOR pathway triggered by *SH2D1A* gene mutation. Meanwhile, the IgM level of the patient was significantly higher than the normal reference value, indicating activation of PI3K-AKT-mTOR signaling pathway might be the potential pathogenic mechanisms of XLP-1. In general, phosphorylation of AKT can directly cause the activation of mTOR, but in this study, we first reported that mTOR was inactivated though PI3K and AKT were activated in the parents of proband with healthy phenotype. The specific reason behind this is unknown yet, there were published studies indicated that mTOR may be activated independently of PI3K in both T and B cells,^[29]^ which might explain the inactivation of mTOR. In addition, studies of PI3K mutant mouse models have shown that at the absence of p85α or p110δ, GC formation was impaired, proliferation and differentiation of mature B cells was decreased, and humoral immunity showed a cliff decline.^[18]^ As for B cell subsets, the proportion of follicular B cells was less than 50%, while proportions of marginal zone B and B1 cells were increased sharply.^[30]^ Follicular B cells are mainly developed into plasma cells, which can produce a large number of antibodies with high affinity. Marginal zone B cells can quickly recognize T1 and TD antigens through their surface BCR and produce antibodies with low affinity. B1 cells contribute to autoantibody reactions and antibody polyreactions and can also produce a large number of antibodies with low affinity including natural antibody IgM and mucosal antibody IgA. Overall, excessive activation of PI3K signal can inhibit the proliferation and differentiation of B cells and lead to the decrease of humoral immunity, which may be the potential reason for the onset of disease in our study. The molecular pathogenic mechanisms of XLP-1 may involve other types of lymphocytes and their internal signaling pathways which needs to be explored by more studies. Also, the correlation and interaction patterns of SAP and PI3K pathways still need to be clarified by further studies.

XLP-1 presents in patients at an average age of 2.5 years, but the proband in this study was only 7 months old at onset. Such a young age with severe liver injury and HLH was very rare in previously reported XLP-1 cases, thus greatly increasing the difficulty of diagnosis and treatment. The only effective treatment for XLP-1 at present is HSCT. However, HSCT treatment requires fulfillment of strict matching and screening criteria and comes with a high surgical risk, so it is not suitable for all XLP-1 patients. In addition to HSCT, gene therapy has developed rapidly in recent years in animal models and is expected to be applied in the treatment of human gene-deficient diseases including XLP-1 in the future.

## 5. Conclusions

In this study, we provided a detailed description of the clinical features of an XLP-1 patient and detected that the proband was caused by a hemizygous mutation c.96G > T in SH2D1A gene resulting in a missense substitution of Arginine to Serine (p.R32S). The mutant protein contained a hydrogen bond turnover at the site of mutation and the mutation resulted in downregulation of SH2D1A encoded protein SAP. The PI3K-AKT-mTOR signaling pathway was fully activated in XLP-1 patients, but it was inactive or only partially activated in healthy people or HLH patients, implying that the PI3K-AKT-mTOR signaling pathway may play a role in XLP-1 pathogenesis. This study would be helpful for subsequent research related to the diagnosis and pathogenesis of XLP-1.

## Acknowledgments

The authors sincerely thank the patient and his family members for their participation and support.

YW, YW, WL, and LT contributed equally to this work.

## Author contributions

**Conceptualization:** Yanchun Wang, Yan Wang, Weimin Lu, Lvyan Tao.

**Data curation:** Yang Xiao, Yuantao Zhou, Xiaoli He.

**Formal analysis:** Yanchun Wang, Yan Wang, Weimin Lu, Lvyan Tao.

**Investigation:** Yang Xiao, Yuantao Zhou, Xiaoli He.

**Methodology:** Yanchun Wang, Yan Wang, Weimin Lu, Lvyan Tao.

**Resources:** Yanchun Wang, Yan Wang, Weimin Lu, Lvyan Tao.

**Supervision:** Yu Zhang, Li Li.

**Visualization:** Yanchun Wang, Yan Wang, Weimin Lu, Lvyan Tao.

**Writing – original draft:** Yu Zhang, Li Li.

**Writing – review & editing:** Yu Zhang, Li Li.
